# Beyond the Patient’s Report: Self-Reported, Subjective, Objective and Estimated Walking Disability in Patients with Peripheral Artery Disease

**DOI:** 10.3390/diagnostics11111991

**Published:** 2021-10-26

**Authors:** Nicola Lamberti, Lorenzo Caruso, Giovanni Piva, Luca Traina, Valentina Ficarra, Paolo Zamboni, Vincenzo Gasbarro, Fabio Manfredini

**Affiliations:** 1 Department of Neuroscience and Rehabilitation, University of Ferrara, Via Luigi Borsari 46, 44121 Ferrara, Italy; nicola.lamberti@unife.it (N.L.); lorenzo.caruso@unife.it (L.C.); 2 PhD Program in Environmental Sustainability and Wellbeing, University of Ferrara, Via Paradiso 12, 44121 Ferrara, Italy; giovanni.piva@unife.it; 3 Unit of Vascular and Endovascular Surgery, University Hospital of Ferrara, Via Aldo Moro 8, 44124 Ferrara, Italy; l.traina@ospfe.it (L.T.); v.ficarra@ospfe.it (V.F.); vincenzo.gasbarro@unife.it (V.G.); 4 Vascular Diseases Center, Department of Translational Medicine for Romagna, University of Ferrara, Via Luigi Borsari 46, 44121 Ferrara, Italy; zambo@unife.it; 5 Department of Medical Sciences, University of Ferrara, Via Fossato di Mortara 64, 44121 Ferrara, Italy; 6 Unit of Rehabilitation Medicine, University Hospital of Ferrara, Via Aldo Moro 8, 44124 Ferrara, Italy

**Keywords:** peripheral artery disease, exercise testing, claudication, reliability, diabetes

## Abstract

Among patients with peripheral artery disease, an altered estimation of walking ability reported to the physician may influence the choice of treatment. We compared claudication distance (CD) values reported by patients or assessed by validated protocols to elaborate a formula capable of estimating more reliable values. Three hundred fifty-nine patients with claudication were measured at the time of entry into a rehabilitation program. Walking performance was obtained by patients’ reports (self-reported claudication distance, SR-CD) and was directly assessed to determine the claudication and maximal walking distance by the 6-min test (6-CD and 6-MWD) and an incremental treadmill test (T-CD and T-MWD). The degree of muscle deoxygenation was objectively determined at the calf by near-infrared spectroscopy (NIRS) during the treadmill test. Among the 289 subjects analyzed, SR-CD exceeded both 6-CD and T-CD (+155 and +182 m, respectively). SR-CD was moderately correlated with T-CD (r = 0.30), 6-CD (r = 0.32), and 6-MWD (r = 0.29) but not with muscle deoxygenation per meter walked, unlike T-CD and 6-CD. A formula adjusted for the presence of diabetes reduced patient overestimation by 92%. The patient’s reported claudication distance was generally overestimated compared to objective measures, and it was made more reliable through a corrective factor for easy use in a clinical setting.

## 1. Introduction

Peripheral artery disease (PAD) is a widespread condition that is highly prevalent in elderly individuals and may significantly limit functional capacity [1]. Up to one-third of PAD patients report vascular claudication or limb pain or discomfort while walking, with the need to slow down or stop for a variable length of time [1,2,3].

In the intermediate stages of the disease when these symptoms are reported, optimal medical therapy, exercise, and/or revascularization represent advised interventions for disease management [1,4,5]. However, particularly for the last two options, patients’ perceived limitations and expectations in terms of quality of life changes, on the one hand, and surgeons’ evaluations in terms of risks and benefits, on the other hand, play a role [1].

From this perspective, knowledge of the degree of walking limitation is relevant. Although walking ability can be assessed by questionnaires and validated outcome measures tests [1,4,5], patients’ self-reported limitation often represents the crucial element involved in the decision-making process [6]. This fact may affect both the staging of the patient and the outcome assessment following a possible treatment [7,8]. Previous studies have evaluated the reliability of patients’ responses by comparing the report of walking autonomy with data estimated by validated questionnaires or assessed by laboratory- or community-based walking tests [6,9,10,11,12,13,14,15], with mixed results. These studies were performed in a limited number of patients and often compared the individual report with data collected by validated outcome measures, which were also potentially affected by subjective perception [15]. Unfortunately, physiological, pathological, and training-related factors [12,16,17,18] may individually alter pain perception [6,10,11,13]. However, technology may enable objective assessment of the limitation during outside walking in a “real-life” situation [9,13,15,19] or quantification by standardized laboratory tests of the critical metabolic factors involved in ischemic pain and associated with the handicap [20,21]. In particular, near-infrared spectroscopy (NIRS) has made possible the noninvasive study of local muscle metabolism and the objective and dynamic quantification of the degree of muscular deoxygenation in individuals, including PAD patients [8,22,23,24,25]. This objective parameter might offer further information when compared with patient reports and with traditional walking tests based on patients’ subjectivity.

In a real-world situation, we studied a large population of PAD patients complaining of vascular claudication with varying severity, aiming (i) to verify the reliability of the self-reported claudication when compared with values directly assessed by ground and treadmill walking protocols and with the degree of muscle oxygenation objectively measured at the calf by NIRS and (ii) to narrow down the possible discrepancy between self-reported and measured values by a simple formula that can be easily applied in a clinical setting to support the decision-making process.

## 2. Materials and Methods

This is a cross-sectional study involving patients with PAD and vascular claudication referred to the vascular rehabilitation program at the Unit of Rehabilitation Medicine at University Hospital of Ferrara. The CE-AVEC Ethics Committee approved the study (277/19).

### 2.1. Participants

All patients referred to the rehabilitation program from January 2017 to December 2020 were screened to take part in the study. The inclusion criteria for the present analyses were: males and females aged >18; PAD with claudication at Rutherford’s stage I-III previously diagnosed in the Unit of Vascular Surgery of the University Hospital of Ferrara; absence of a documented cognitive impairment; and ability to walk on a treadmill. Patients were excluded from the analyses if they reported a claudication distance >500 m.

### 2.2. Measurements Collected

Self-reported claudication distance (SR-CD). All patients were asked to estimate their claudication walking distance by the following question: “What is the distance (in meters) you can walk at your usual pace on a flat surface before you experience the onset of leg pain?”.

Corridor walking distance. All patients performed the 6-min walking test according to published standards [26]. Patients were instructed to cover as much distance as possible in 6 min by walking back and forth in a 21-m corridor. Patients were asked to report the onset of symptoms (recorded as 6-CD, corresponding to pain-free walking distance), and they were allowed to rest when necessary. The total distance walked (6-MWD) was also collected.

Treadmill walking test. For the measurements of walking capacity, a validated incremental treadmill protocol was employed [27]. After familiarization with the instrument by walking a 1-min warm up, patients performed the level ground test that started at an initial speed of 1.5 kmh^−1^ and was progressively increased by 0.1 km^−1^ every 10 m until reaching the maximal speed attainable, as limited by symptoms or fatigue. The treadmill claudication distance (T-CD) and maximal walked distance (T-MWD) were recorded.

Objective determination of muscle deoxygenation by near-infrared spectroscopy (NIRS). During the previously described incremental treadmill test, each patient was equipped with a continuous wave near-infrared spectroscopy (NIRS) system (Oxymon MK III Artinis Medical System, Elst, The Netherlands), consisting of two channels using intensity-modulated light at 1 Hz frequency and 3 wavelengths (905, 850, and 770 nm) corresponding to high absorption of oxyhemoglobin (oxy) and deoxyhemoglobin (deoxy). Near-infrared light propagating through biological tissue is partly absorbed or scattered by the tissues and partly recollected by the detector; therefore, the intensity of the recollected light provides information about oxy- and deoxyhemoglobin concentrations. After taking skinfold measurements, NIRS optodes with an interoptode distance of 3.5 cm, allowing light penetration of approximately 20 mm, were placed on the medial side of the calf (gastrocnemius muscles) and secured with tape.

At the end of the walking test, semiquantitative data collected by the NIRS instrument were analyzed using Oxysoft 2.0.47 software. To quantify the variations, after assigning the first value (beginning of the test) of each hemoglobin trace test to be 0, the area under the curve (AUC) for each variable was calculated by summing all the single values obtained until the end of the test, as validated [24]. The parameters considered were the AUCs of oxy- and deoxyhemoglobin that were calculated for the more diseased limb of each patient as determined by the lower ABI value, or the more diseased limb according to the duplex ultrasound examination in the case of incompressible vessels. To avoid possible bias related to the duration of the treadmill test, the degree of deoxygenation per meter was calculated by dividing the AUC of oxyhemoglobin by the T-MWD covered during the treadmill test. This calculated parameter was employed for the subsequent analyses.

Hemodynamics. The ankle-brachial index (ABI) was measured according to published standards [5] using a Doppler ultrasound transducer (Dopplex SD2, Huntleigh Healthcare Ltd. Diagnostics, Cardiff, UK) and a standard blood pressure cuff. The vessels were considered “not compressible” for ABI measurements >1.31 or for a procedure that had been interrupted due to painful symptoms at a cuff pressure of 300 mmHg with a Doppler signal still present.

Demographics, anthropometrics, and clinical data. At the entry visit for each patient, the following parameters were collected: age, height, and weight (for BMI calculation), risk factors (derived from use of medications or clinical examinations), comorbidities with calculation of the Charlson Index, history of PAD (duration of disease, previous interventions), and type and location of endovascular lesions.

### 2.3. Statistical Analysis

Data distribution was verified with a Shapiro-Wilk test. Comparisons between SR-CD and measured distance, considering both walking tests, were performed with Bland–Altman plots and Passing and Bablok regressions. Correlations between estimated and walked distances and objective measurement of muscle oxygenation by NIRS were assessed by a Spearman rank test.

A multiple regression model selecting 6-CD as the dependent variable with a stepwise method of selection was used to determine the potential impact of independent variables (including SR-CD, anthropometrics, risk factors, comorbidities, ABI, etc.) on the actual measured distance.

In addition, a logistic regression model was employed to determine the impact on a discrepancy between 6-CD and SR-CD (dependent variable, dichotomized as > or < of 100 m) of the previously mentioned independent variables, properly dichotomized when necessary. A *p* value < 0.05 was considered statistically significant. Statistical analysis was performed with MedCalc^®^ Statistical Software version 20.011 (MedCalc Software Ltd., Ostend, Belgium).

## 3. Results

Three hundred fifty-nine patients were measured at the time of entry into the rehabilitation program. Within this group, 70 patients were excluded for not matching the inclusion criteria, in particular for reporting a claudication distance >500 m (*n* = 57). The remaining 289 patients were then analyzed. The demographic and clinical characteristics of the enrolled patients are reported in Table 1.

### 3.1. Self-Reported and Measured Walked Distances

At baseline, patients reported an SR-CD of 264 ± 114 m.

During the 6MWT, 171 patients (59%) needed to stop during the test. The total 6-MWD covered was 305 ± 83 m, whereas the 6-CD was 136 ± 82 m. The T-CD and T-MWD were 110 ± 85 and 172 ± 92 m, respectively.

Patients’ SR-CD was significantly correlated with all the measured parameters, with rho values ranging from 0.25 to 0.32. The data are reported in Table 2.

### 3.2. Comparison between Self-Reported and Measured Walked Distance

All Bland–Altman plots conducted comparing estimated distance and actual distance rejected the null hypothesis or confirmed a significant difference between SR-CD and actual measurement. In particular, differences from the estimated distance were: 155 m (95% confidence interval (CI) 141–168 m; *p* < 0.001) for 6-CD and 182 m (95% CI 169–196 m; *p* < 0.001) for T-CD measured on the treadmill.

**Figure 1:**
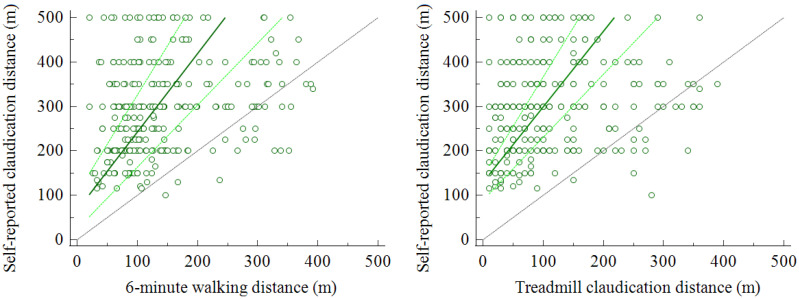
Passing and Bablok regressions showing significant deviation from linearity (*p* < 0.001) between self-reported claudication distance and measured walking distance. Legend: dark green, regression line; light green, 95% confidence interval; grey line, identity line.

Passing and Bablok regressions confirmed the significant deviation from linearity for all four parameters considered, with the majority of points located in the upper-left half of the diagram, indicating an overestimation of the SR-CD compared to the actually measured SR-CD. Data comparisons for 6-CD and T-CD are reported in Figure 1.

### 3.3. Relationship with Objective Measurements

Patients showed a mean AUC of oxyhemoglobin at the calf of the more diseased limb of −2.008 ± 1.902 arbitrary units and a value of deoxygenation per meter of −12.2 ± 9.4 arbitrary units.

**Figure 2:**
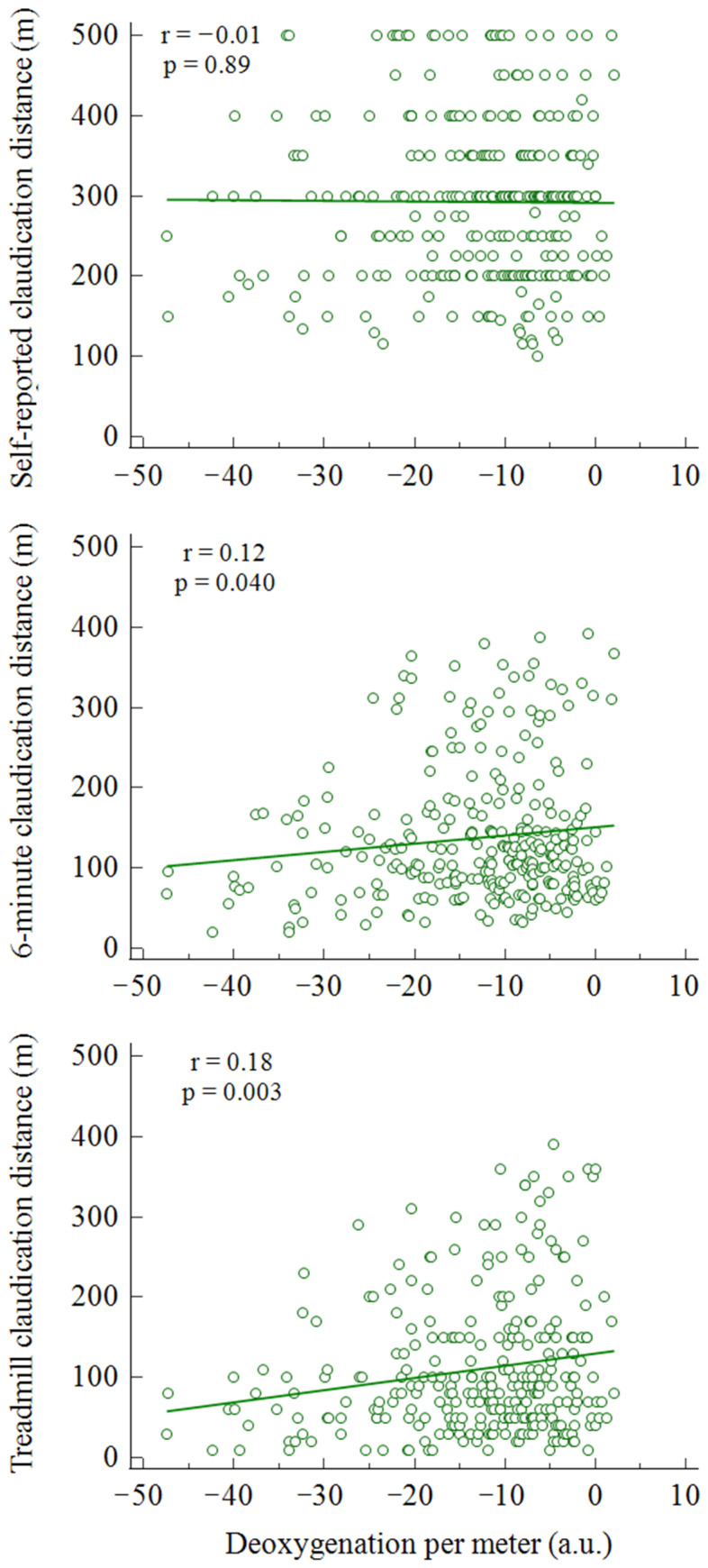
Rank correlations between deoxygenation per meter and SR-CD, 6-CD, and T-CD. Legend: green line represent the correlation line.

Significant relationships between muscle oxygenation and deoxygenation per meter were observed with 6-CD (r = 0.12; *p* = 0.040) and T-CD (r = 0.18; *p* = 0.003), but not with the patient’s SR-CD (r = −0.01; *p* = 0.89) (Figure 2).

### 3.4. Factors Related to Walked Distance

A significant multiple regression model (R^2^ = 0.09; *p* < 0.001) considering 6-CD as the dependent variable identified only SR-CD as an independent factor (partial r = 0.28). All the other independent variables were not included, namely, age, BMI, Charlson Index, ABI, and duration of PAD. The obtained equation was 6-CD = 70.7 + (0.22 × SR-CD).

A subsequent logistic regression model using a difference between estimated distance and 6-CD >100 m (dichotomized as “1”) as the dependent variable was found to be significant (R^2^ = 0.10; *p* < 0.001). The only independent variable retained in the model was the presence of diabetes (odds ratio 2.48, 95% CI 1.41–4.34).

Therefore, the previously performed multiple regression model was repeated for the two populations of patients with and without diabetes. The two equations obtained from significant models were inserted into a system of equations, which resulted in the following formula for the estimation of the walked distance (Equation (1)):

6-CD (m) = 78.6 + (0.18 ∗ SR-CD) − [diabetes × 11.0)](1)

**Figure 3:**
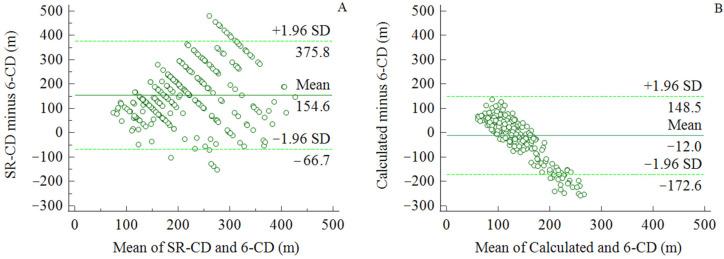
Bland–Altman plots comparing SR-CD and 6-CD (**A**) versus 6-CD and calculated distance through the proposed formula (**B**). Graph shows a mean difference of 155 m for SR-CD (**A**) and 12 m for calculated CD (**B**).

The Bland–Altman plots comparing the actual 6-CD with the calculated distance with the proposed formula showed a mean difference of 12 m (95% CI 2–24 m) (Figure 3).

## 4. Discussion

This study, carried out in a large population of PAD patients with claudication, confirms the low reliability of the patient in reporting his walking ability by comparisons with both validated tests and with an objective NIRS-based parameter of muscle oxygenation during walking. The study also provides a rapid system for converting the overestimated data reported by the patient into a mathematically congruent measure for possible clinical use.

The issue of the walking disability perceived by the patient with intermittent claudication and reported to the physician, as well the necessity of reliable information to guide therapeutic decisions (e.g., involving invasive treatments), is an old matter of discussion [3]. In general, patient’s estimation was found to have variable reliability in studies based on comparisons with validated questionnaires or tests or with an objective assessment of disability through technological devices [3,6,9,13,15]. These various methods have in turn been compared with each other by several authors [6,9,10,13,15,28]. If an overestimation from 20% to 50% of walking ability compared to the measured values was observed [3,7,13], an underestimation was also found [9,11,15], or a certain comparability [6], or, in addition, variability according to the PAD population examined (e.g., with or without diabetes) [8,10,12].

The present data highlight a significant overestimation of the data for walking ability reported. The possible difference from other studies may derive from the sample size of the present study, which is larger than other observations, and consequently of the characteristics (i.e., age, sex, functional capacity and different comorbidities) of the population involved.

The study shows a quite similar quantitative relationship between patients’ reports and the actual measurements. The relationship between the SR-CD and the 6-CD and T-CD was low-moderate (r = 0.32 and 0.30, respectively), with the corridor test showing the higher correlation, as previously observed [6]. This aspect is of interest considering the reported usefulness of the 6-MWD for routine clinical evaluation of walking capacity in claudication patients [11].

A further aspect to highlight compared to previous studies is that the individual values collected with both measures (corridor and treadmill) correlated with the only data objectively measured as the NIRS unit of deoxygenation per meter walked on the treadmill. Even if an albeit weak correlation with a muscular metabolic state was observed, no relationship with self-reported data was found (Figure 2). In the present study, the use of the NIRS technique allows the inclusion of an objective physiological parameter free from the patient’s perception. In the past, a different measurement with NIRS among the factors related to patient performance in patients with claudication represented a predictor of walking performance [20].

After all, the common aspect linking the observations is the individual variability between the estimated and measured values. In our experience, according to the study population, various factors of different natures may play a role. Among these, behavioral factors related to the perception of the distance traveled may be considered. Some patients do not walk regularly and without landmarks, walk with their dog with imposed breaks, or regularly walk in habitual paths of known length. The usual individual walking speed [10], which can also vary by 20–40% between subjects according to our experience, can also influence the report and the willingness to insist on symptoms that differ from subject to subject [11]. In addition, the discrepancy with measured values could also derive from a modified walking pattern in the course of evaluation, with the risk of anticipating or delaying the onset of symptoms. Finally, the walking capacity report may be affected by the daily variability of walking efficiency or by age-related cognitive problems and the related influence from the caregiver present at the interview. However, it is known that sensory disorders of the lower extremities may alter pain perception [8,12] and pain tolerance, possibly associated with repeated bouts of walking in the presence of moderate–intense pain, as generally recommended [29].

Moreover, the presence of diabetes may influence the performance, as seen in the self-reported walking distance in a treadmill test [30]. In addition, the presence of peripheral neuropathy related to diabetic pathology could reduce the perception of ischemic symptoms, paradoxically favoring walking autonomy. In a study with NIRS, PAD subjects with diabetes walked the same distance as patients without diabetes, even in the presence of a 2.5-fold greater degree of muscle deoxygenation [8].

Finally, beyond the limitations of the PAD assessment [31], the protocol used in the comparison with the reported distance can play a role in relation to the structured context. The necessity of complying with predefined parameters (e.g., a fixed or increasing imposed speed or slope in a treadmill test) makes it impossible to implement strategies, such as slowing their pace (even involuntarily) at the very early symptoms of fatigue.

Even with these possible limitations, in view of the difficulties patients exhibit in perceiving one’s own walking disability, the functional capacity should be measured with standardized tests to define the need for intervention considering the risk and social costs related to this choice or for rehabilitative exercise. In the event that it is impossible to have an exercise physiologist present for tests or a trained person in charge of function measurements by means of a questionnaire, which is also time-consuming, the use of a rapid calculation may estimate, with greater reliability, the real functional state of the subject. Previous studies suggested age or individual velocity as critical factors [10,12]. In our study, through a combination of regression models, we found that only the presence of diabetes represented a significant corrective factor to better fit the overestimated patient report. This fact is interesting in itself considering that in patients with PAD and diabetes, this poor reliability is coupled to other critical issues, such as the poor immeasurability of ABI reducing the accuracy of monitoring [32] or the risk of a sudden clinical worsening into Fontaine’s stages 3 and 4. The calculation allows a better estimation of the walking disability, as shown by Figure 3.

The present study has limitations with regard to the data collection in terms of self-reported walking ability. Indeed, this parameter was not collected through the use of validated tools (i.e., a questionnaire or structured interview) but through a standardized question asked to the patient during the first visit, considering that patients preferably reported only a single walking distance, which usually corresponds to the time of the first onset of symptoms [15]. In addition, the relationships with the NIRS parameter were calculated on the more impaired limb, considering that in bilateral disease, a sum of the metabolic effects may affect the 6-MWD.

We indicate as a strength of the study the sample size analyzed in a real-world situation, which allows us to draw conclusions generalizable to a PAD population with comparable characteristics. The use of tests with different protocols (incremental to exhaustion or time-based) and with subjective and objective parameters obtained is of further interest, particularly when employing a technological objective metabolic measurement.

## 5. Conclusions

The present study confirmed the poor reliability of the self-reported data by revealing that most patients with claudication overestimate their walking performance, despite the variability of the appearance or perception of symptoms. While waiting for the implementation of technological wearable devices [9,13,15,19] in clinical practice, the study offers a quick system of conversion by the obtained formula from the data reported by the patient to more likely data, considering that the present information could be of interest in supporting clinicians in decision-making choices.

## Figures and Tables

**Table 1:** Characteristic of the population included in the study.

	Analyzed (*n* = 289)
Age (years)	71 ± 9
Body mass index (kgm^−2^)	25 ± 6
Males, *n* (%)	225 (78)
Risk factors, *n* (%)	
Smoking	254 (88)
Current smoking	72 (25)
Hypertension	248 (86)
Hyperlipidaemia	208 (72)
Diabetes	156 (54)
Chronic kidney disease	52 (18)
Comorbidities, *n* (%)	
Coronary artery disease	87 (30)
Cerebrovascular disease	14 (5)
Osteoarticular disease	75 (26)
Rheumatic diseases	12 (4)
Chronic-obstructive pulmonary disease	15 (5)
Age-adjusted Charlson Comorbidity Index	6 ± 2
Peripheral artery disease	
Disease duration (years)	6 ± 5
Lower limb revascularization	86 (27)
ABI more affected limb	0.63 ± 0.22
ABI less affected limb	0.83 ± 0.19

Abbreviations: ABI, ankle-brachial index.

**Table 2:** Correlations between self-reported and actual walking distances.

	SR-CD	6-CD	6-MWD	T-CD	T-MWD
SR-CD	-	0.319 <0.001	0.291 <0.001	0.304 <0.001	0.254 <0.001
6-CD	0.319 <0.001	-	0.560 <0.001	0.592 <0.001	0.496 <0.001
6-MWD	0.291 <0.001	0.560 <0.001	-	0.512 <0.001	0.689 <0.001
T-CD	0.304 <0.001	0.592 <0.001	0.512 <0.001	-	0.739 <0.001
T-MWD	0.254 <0.001	0.496 <0.001	0.689 <0.001	0.739 <0.001	-

Abbreviations: SR-CD, self-reported claudication distance; 6-CD, 6-min claudication distance; 6-MWD, 6-min walking distance; T-CD, treadmill claudication distance; T-MWD, treadmill maximal walking distance.

## Data Availability

The dataset of the study is available upon request at nicola.lamberti@unife.it.
